# Summering

**Published:** 1868-05

**Authors:** 


### SUMMERING.

The Editor of the "New York Evening Mail" is preparing a book, of which many copies will be demanded, if gotten up properly; it should show how short and long excursions can be made out of New York, and at what cost; also, where families can spend the summer pleasantly, how to get there, and at what rates, from two dollars a week each, up to two hundred; the rich can take care of themselves ; but the struggling want to know where cheap, plain, healthful and enjoyable accomodations can be had. It would be a good plan for our country friends to advertise all these things liberally and truthfully in Mr. Sweester's book ; especially the prices and modes and time of reaching their places. If you want the luxury of rest, and that is what our citizens most need, go to a farm-house, several miles from a railroad station, or steamboat landing, which promises nothing more than cleanliness and plain fare, whose occupants make no pretensions to have descended from the Great Mogul; or to have been once rich, and lost all by going security ; who don't own to having any rich relations, nor to have had as boarders, formerly, the Queen of Sheba, or Cleopatra, or Victoria Regina. If you happen at a farm-house where they begin on "this line," you may be sure that will be their only "line," and their biggist line, to the end of the chapter, and had better "move" right off, bag and baggage, even if it is dark, and camp out that ni ht in the corner of a fence, a mile off. Why do our people submit so passively to the robberies and impositions of Long Branch, Saratoga and Cape May? What of rest, enjoyable rest, do any of such places give to a wearied body, to an over-worked brain, and a constitution ready to break by the excessive toils of city life. Go, reader, where you can dress in your commonest clothing; and you poor over-worked wife don't take that everlasting "sewing" along with you; resolve not to take a single stitch during your entire absence; let your only "sewing apparatus" consist of a ball of yarn, a skein of silk, and two needles; leave your thimble at home; if you sew on all the buttons, and keep the stockings darned, that will be quite enough. Sleep from dark until sunrise; be out and about on foot or horse, in carriage, rowing, boating, swimming, botanizing; or with hammer and microscope study the rocks and read the histories of the localities from ages before the Flood, as you can do; climb mountains, explore caves; in short, do anything and everything which will entertain, instruct, or gratify yourself, or others, from breakfast until noon, and from dinner to sun-down; so that when you come home to dinner, you will be as hungry as a bear, and bacon, cabbage and corn-bread will taste sweet to you; after dinner you go again, that when you come home at sun-down, you will sigh for the bed, hardly taking time to have a dry cracker and a cup of tea, all that you ought to have, and all that night you will sleep like a baby, and next morning you will feel hungry enough to eat your wife and children, and so happy and good natured that life would have new charms for you. Rest from care, from business anxiety, from dress, and the needle, from the conventionalities of society, these are what city people need during summer, and they most certainly are not to be had at the seaside or the Spa.

A grand good idea for all concerned, would be for the managers of the Pacific Railroad to take persons for half fare to the utmost limit of their line, beyond even Cheyenne; this would make the road talked about, would bring it to the attention of the public, who, when they saw the safety and profitableness of the investment, would want more stock than the company want to part with, and would give excursionists a more correct idea of the vastness of our country than they could get by a year's reading. At Cheyenne we could almost shake hands with Brother Brigham and his—by the way, wasn't it he, or him, with whom Beecher fraternizes with so cordially, or was it Chapin? we know it was some outsider; at all events, it would be a grand and a novel excursion; what's the use of hanging always around the Falls, and Mammoth Cave, and the Bay of Fundy; let us get up some new thing under the sun, and by next year we may be able to "rail" it to the Yosemite, the Sierre Nevada, and the cedars of three thousand years ago. Meanwhile, Mr. Sweester, get your book out by the 1st of May, so that we may make a selection for our summering. But alas! the summer of our life is over; its smiles, its sunshines, and its joys are gone; it was not so a year ago; it is for the little ones we are concerned now, and the only life left to us is in their future, may it be one of brightness and health, and purity; the happifying of the world they live in.

